# 

**Published:** 1897-06

**Authors:** 


### CONTENTS.

ORIGINAL ARTICLES: PAGE
The Spaying of Mares. By W. L. WILLIAMS, V.S. . . . . . . . . 355
Silos and the Value of Ensilage. By REV. T. D. DETRICH . . . . . . 362
The Pleasure of Being a Veterinarian. By J. C. MICHENER, V.S. . . . . 365
Acute Laminitis. By B. L. CLARK, M.D.C. . . . . . . . . . 366
Actinomycosis. By B. F. KAUPP, D.V.S. . . . . . . . . . . 371
The Presidential Address, Wisconsin Society of Veterinary Graduates. By G. ED. LEECH . . . . . . . . . . . . . . 373
Tick-fever. By D. F. LUCKEY . . . . . . . . . . . 379
Azoturia. By JOHN R. HART, V.M.D. . . . . . . . . . . 382

ABSTRACTS FROM FOREIGN JOURNALS:
The Marking of the Domestic Animals, and the Use of the Patent Marking-tongs 387

REPORTS OF CASES:
The Intravenous Injection of Artificial Blood-serum for the Relief of Toxæmia and Extensive Hemorrhage. By THOMAS V. SIMPSON, V.S. . . . . . 390
The Operation of Arytenectomy Assisted by the Electrical Lamp. By W. H. ARROWSMITH, D.V.S. . . . . . . . . . . . . . 392

EDITORIALS:
Stick at It, Fellow Veterinarians . . . . . . . . . . . 394
Something for the Consideration of Faculties . . . . . . . . 394
A Peculiar Experience . . . . . . . . . . . . 395
Tennessee Joins Illinois . . . . . . . . . . . . 395
A Much-needed Organization . . . . . . . . . . . 396

MARRIAGES—NECROLOGY—CORRESPONDENCE . . . . . . . 397
LEGISLATION . . . . . . . . . . . . . . 402
BOOK REVIEWS . . . . . . . . . . . . . . 405

COMMENCEMENT EXERCISES:
Kansas City Veterinary College . . . . . . . . . . . 409

SOCIETY PROCEEDINGS:
Veterinary Medical Association of New York County—Keystone Veterinary Medical Association—The Veterinary Medical Society of the State of New Jersey and Vicinity—Chicago Veterinary Society—Massachusetts Veterinary Association—Organization of St. Louis Veterinarians—State Boards of Veterinary Medical Examiners—Association News—Resolutions Passed by the Chicago Veterinary Society—U. S. V. M. A. . . . . . . . . 410–418

SOMETHING TO INTEREST EACH READER—PERSONALS—GLEANINGS, 418–422

